# Analysis of Factors Contributing to the Severity of Large Truck Crashes

**DOI:** 10.3390/e22111191

**Published:** 2020-10-22

**Authors:** Jinhong Li, Jinli Liu, Pengfei Liu, Yi Qi

**Affiliations:** 1 School of Mathematics and Statistics, Qilu University of Technology (Shandong Academy of Sciences), University Road 3501, Jinan 250353, China; lijinhong@qlu.edu.cn; 2 Department of Transportation Studies, Texas Southern University, 3100 Cleburne Street, Houston, TX 77004-9986, USA; j.liu5684@student.tsu.edu; 3 Department of Civil and Environmental Engineering, the University of North Carolina at Charlotte, EPIC Building, Room 3366, 9201 University City Boulevard, Charlotte, NC 28223-0001, USA; pliu11@uncc.edu

**Keywords:** large truck crash, injury severity, random forest, gradient boost decision tree, AdaBoost, mixed logit model, contributing factors, AK level crashes

## Abstract

Crashes that involved large trucks often result in immense human, economic, and social losses. To prevent and mitigate severe large truck crashes, factors contributing to the severity of these crashes need to be identified before appropriate countermeasures can be explored. In this research, we applied three tree-based machine learning (ML) techniques, i.e., random forest (RF), gradient boost decision tree (GBDT), and adaptive boosting (AdaBoost), to analyze the factors contributing to the severity of large truck crashes. Besides, a mixed logit model was developed as a baseline model to compare with the factors identified by the ML models. The analysis was performed based on the crash data collected from the Texas Crash Records Information System (CRIS) from 2011 to 2015. The results of this research demonstrated that the GBDT model outperforms other ML methods in terms of its prediction accuracy and its capability in identifying more contributing factors that were also identified by the mixed logit model as significant factors. Besides, the GBDT method can effectively identify both categorical and numerical factors, and the directions and magnitudes of the impacts of the factors identified by the GBDT model are all reasonable and explainable. Among the identified factors, driving under the influence of drugs, alcohol, and fatigue are the most important factors contributing to the severity of large truck crashes. In addition, the exists of curbs and medians and lanes and shoulders with sufficient width can prevent severe large truck crashes.

## 1. Introduction

Since 1994, Texas has had the highest number of fatal crashes involving large trucks in the U.S. [1].Among these large truck crashes, the AK level crashes (A is the incapacitating crash, and K is the fatal crash) often result in immense social and economic losses. To prevent and mitigate the occurrence of AK level truck crashes, factors that significantly contribute to such severe crashes need to be identified before appropriate countermeasures can be explored. This study was to investigate the risk factors that contribute to the occurrence of AK level truck crashes. Although a significant number of crash severity studies have been conducted, few have focused on the AK level truck crashes [2,3].

From a methodological perspective, a wide spectrum of modeling approaches has been adopted in the crash severity analysis. Both traditional regression models (such as binary logit model, nested logit model, and mixed logit model) and the machine learning (ML)-based methods (such as random forest, adaptive boosting, gradient boost decision tree, neural network, and support vector machine) have been applied for the crash severity analysis. The traditional regression models and ML-based methods have their own advantages and limitations, which are introduced in detail in the literature review section. These models’ capabilities in analyzing the impacts of contributing factors to server truck crashes need to be investigated. 

This study was to investigate the contributing factors to the AK level large truck crashes by using both classification tree-based ML methods and regression models. For this purpose, three different types of classification tree-based ML methods, including random forest (RF), adaptive boosting (AdaBoost), gradient boost decision tree (GBDT), were used for identifying and analyzing the factors that have significant impacts on the severity of large truck crashes. In addition, a mixed logit model was developed for serving as a benchmarking model for comparison. The results of the four different types of models were compared and evaluated based on the identified factors and their impacts. The results of this study indicated that the GBDT method outperforms the other two types of ML methods (i.e., RF and AdaBoost). Besides, the GBDT method can effectively identify both categorical factors and factors, and the directions and magnitudes of the impacts of the factors identified by the GBDT model are all reasonable and explainable. 

Previous studies have investigated the factors that affect the severity of crashes with a variety of focuses. Some studies have focused on certain types of crashes, such as rear-end crashes or rollover crashes, and others have focused on certain locations where the crashes occurred. In terms of methodologies, some studies have used traditional regression models, and some have used ML-based methods. For the traditional regression models, logit models and ordered probit models have been widely used for crash severity analysis. For example, Chang and Mannering (1999) used nested logit models to analyze the severity of injuries for both truck-involved crashes and non-truck-involved crashes [4]. Khattak et al. (2003) used ordered probit models to identify the contributing factors, and the focus of their study was the large truck rollover crashes [5]. 

Besides the traditional logit models and ordered probit models, some advanced regression modeling techniques have been explored by previous studies. For example, Xie et al. (2009) conducted a motor vehicle crash injury severity analysis using Bayesian ordered probit (BOP) models [6]. Pahukula et al. (2015) utilized random parameter logit models to examine the impacts of time of day on the injury severity of large truck-involved crashes [7]. Al-Bdairi and Hernandez (2017) used an ordered random parameter probit model to analyze the injury severity of large truck-involved run-off-road crashes in Oregon [8]. Ahmed et al. (2018) explored the contributing factors to the large truck-involved crashes on rural highways in Wyoming using Bayesian binary logit models [9].

For ML-based techniques, the techniques applied to traffic safety studies include classification tree-based models, neural networks, and support vector machine models. In recent years, the classification tree-based ML methods have been widely employed for crash risk prediction and identification of contributing factors [10,11]. A classification tree-based ML method decides which crash risk factors should be chosen as the decision nodes and which features can provide more information or reduce more uncertainty about the severity of traffic crashes based on information gain and entropy. Chang and Chien (2013) used the classification and regression tree (CART) method to examine the impacts of the driver and vehicle-related factors on the severity of injuries in large truck crashes [12]. The importance of factors was analyzed according to the structure of the developed classification tree. The results showed that drunk-driving is the most significant factor that contributes to the severity of injuries in large truck crashes on the freeways. Yu and Abdel-Aty (2014) focused on developing crash severity analysis models by first selecting the most important variables associated with the severe crash occurrence using the random forest (RF) method [13]. Then, three different types of models (fixed-parameter logit model, support vector machine model, and random parameter logit model) were developed to analyze crash injury severity. In some other studies on crash severity analysis [14,15], the RF method was also used for preselecting the independent variables for the regression models. Zeng and Huang (2014) proposed a convex combination (CC) algorithm to train a neural network (NN) model for crash injury severity prediction and a modified NN pruning for function approximation (N2PFA) algorithm to optimize the NN structure [16]. According to the results of this study, the CC algorithm outperforms the traditional back-propagation algorithm both in convergence ability and training speed. Tang et al. (2019) proposed a two-layer stacking framework to predict crash injury severity [17]. The first layer integrates the advantages of three base classification methods: RF, AdaBoost, and GBDT. The second layer completes the classification of crash injury severity based on a logistic regression model. 

In general, both the traditional regression models and ML-based methods have their own advantages and limitations. The regression models have explicit equations that link the independent variables (risk factors in this study) to the dependent variable (crash severity level); thereby, it has a good capability in analyzing the impacts of independent variables. However, they have difficulties in detecting and interpreting complex or high-order interactions among independent variables [18]. Some ML-based methods, like neural networks, have been known for their strong prediction capabilities. However, they have been criticized for operating like a black box and unable to explicitly explain the impacts of independent variables on the dependent variables [19]. Classification tree-based ML methods like random forest have been known for their relatively better transparency [20]. However, there is still a lack of explicit equations for the developed models. In addition, few studies have been conducted on comparing the classification tree-based ML models with the traditional regression models to make a full scale of understanding of the model’s capabilities in analyzing the impacts of independent variables. This study is to fill these identified gaps in the existing studies.

In this paper, a literature review of previous studies related to the crash injury severity analysis is presented at first, followed by an introduction of the methodology, the collected crash data, and the independent variables considered in the analysis. After that, modeling results and their implications are discussed in detail, which leads to conclusions and recommendations. 

## 2. Collection and Processing

### 2.1. Data Description

A large and comprehensive truck crash database was developed based on the crash data collected from the Texas Crash Records Information System (CRIS). It contained the truck crash records for the entire state of Texas from 2011 to 2015. A large truck, as defined by the U.S. Department of Transportation (DOT), is any truck with a gross weight rating greater than 10,000 pounds. Crashes that involved one or more large trucks were collected for this study. In total, the final dataset contained records of 85,184 large truck crashes and more than 170 attributes for each crash record, including information about the drivers, vehicles, characteristics of the crashes, roadway conditions, and environmental conditions.

### 2.2. Dependent and Independent Variables

The dependent variable in this analysis was the severity level of large truck crashes, and it had only 2 different levels: AK level crashes (y = 1) and non-AK level crashes (y = 0). In total, there were 6.37% of AK level crashes and 93.93% of non-AK level crashes. The independent variables were carefully selected from over 170 attributes of the large truck crash data based on their categories, their correlations between each other, their relationship to the dependent variable, and the quality of the data. 

At first, different types of variables related to the roadway, environment, and driver’s characteristics were derived and classified into different categories, as shown in Table 1. Then, the correlations between these variables were analyzed. Some of these variables were highly correlated. For example, road surface conditions (dry, wet, and ice-covered) and weather characteristics (clear, rain, and snow) were highly correlated factors. To avoid the collinearity problem, the weather characteristic factors were kept in the model, while the surface-condition factors were removed. In addition, most of the independent variables were categorical variables, and they were all converted to the dummy variables, as shown in Table 1. It can be seen that the variables in the same category were highly correlated. Taking the “Lighting Conditions” category as an example, the lighting conditions included the “daylight”, “dark no light”, “dawn”, “dark light”, and “dusk”, which was a complete list, and the lighting condition of a crash must be one of these five conditions. Thus, if we included all these dummy variables, their sum would be equal to 1. To avoid the dummy variable trap, one baseline variable was identified for each category and was excluded from the model [21]. Furthermore, some factors did not have a very clear causal relationship with the dependent variable. For example, the factor “number of lanes blocked by the crash” was not the cause of a severe crash but was determined simultaneously with the crash severity level when a crash occurred. Therefore, this type of variable should also be removed from the model to avoid the endogeneity problem [22]. Finally, by carefully examining all the factors in different categories, only 40 independent variables were finally selected, as listed in Table 1, and the distributions of variables are presented in Table 2. 

## 3. Methodology

As mentioned in the literature review, a variety of regression methods have been applied to analyze crash-severity data, including the traditional regression models, such as the multinomial logit model, ordered logit model, and so on. The mixed logit model is a widely used regression model for crash injury severity analysis. Many studies have found that the mixed logit model could achieve a closer estimation of the crash probabilities to the observations by allowing parameters to differ across observations [14,23,24]. Thus, in this study, the mixed logit model was chosen as the baseline regression model to compare with the ML models. Besides, the classification tree-based ML methods have been widely employed for crash risk prediction and identification of contributing factors [10,11,25]. Note that a number of traffic safety studies have used classification tree-based ML methods for preselecting the independent variables for the mixed logit model to efficiently determine the random parameters and improve model accuracy [13,14]. Therefore, there is a need to find out if the classification tree-based methods can effectively and correctly identify contributing factors that will also be identified by the regression model as significant factors. In this study, three representative tree-based machine learning (ML) techniques, i.e., random forest (RF), gradient boost decision tree (GBDT), and adaptive boosting (AdaBoost), were selected for developing models for identifying the contributing factors to the AK level large truck crashes. For each developed model, the importance of the identified factors was ranked based on the Gini impurity (a criterion to minimize the probability of misclassification). Meanwhile, a mixed logit model was developed as a baseline model for comparing with the developed ML-based classification tree models. Following is the introduction of all the developed models.

### 3.1. Mixed Logit Model

In the development of a mixed logit model for crash injury severity analysis, a utility function needs to be defined, which is a linear function that determines the specific injury severity level i for observation (crash) j:

(1) $$U_{\mathrm{ij}}=\beta_{i}X_{\mathrm{ij}}+\varepsilon_{\mathrm{ij}}$$

where $X_{\mathrm{ij}}$ is the vector of the independent variables, which are the contributing factors to the crash j with severity level i. In this study, there were only two severity levels (i.e., AK crash and non-AK crash). Thus i = 1,2. $\beta_{i}$ is a vector of the coefficients of independent variables, and $\varepsilon_{\mathrm{ij}}$ denotes the error term representing unobservable impacts on the crash outcomes. If $\varepsilon_{\mathrm{ij}}$ is assumed to follow a Gumbel type1 distribution, then the probability of that crash j suffering injury severity level i can be expressed as:

(2) $$P_{\mathrm{ij}}|\beta_{i}=\frac{\exp\left(\beta_{i}X_{\mathrm{ij}}\right)}{\sum_{i=1}^{I}\exp\left(\beta_{i}X_{\mathrm{ij}}\right)}$$

In the mixed logit model, it allows the coefficient vector $\beta_{i}$ to vary across individuals (different crashes). Each element of $\beta_{i}$ may be either fixed or randomly distributed with fixed means, allowing for heterogeneity within the observed crash dataset. By considering the randomly distributed coefficients, the probability of that crash j suffering injury severity level i can be expressed as follows [26]:

(3) $$P_{\mathrm{ij}}=\int \left(P_{\mathrm{ij}}|\beta_{i}\right)f\left(\beta |\varphi \right)d\beta$$

where f(β|φ) is the probability density function (PDF) of the random vector β, and φ denotes a vector of parameters describing the PDF (e.g., the mean and variance of the normal distribution). In this study, it was assumed that all the random coefficients are set to be normally distributed. 

A simulation-based maximum likelihood method is used to estimate the model parameters by using SAS 9.3. It has used a maximum of 100 iterations and 100 simulation points. A backward stepwise variable selection process is conducted. During this process, the random coefficients with insignificant standard deviation are excluded so that the coefficients of those variables are set as fixed. For the final modeling result, the independent variables with both random or fixed coefficients at a significant level of 5% are included. 

### 3.2. Random Forest (RF) 

The RF method combines Breiman’s bagging idea and Ho’s “random subspace method” to construct a collection of decision trees with various sub-samples of the dataset based on information theory [27,28]. Each tree is constructed from a sample drawn with replacement from the training set. A predetermined number of classification trees are generated from the bootstrap sample and combined to give a final prediction. In this study, the model combined classifiers by averaging their probabilistic prediction instead of letting each classifier vote for a single class. The performance of an RF is optimized by minimizing the bias of each tree and the correlations among trees. To minimize the bias, each tree should be grown to maximum depth based on the Gini index [29].

### 3.3. Adaptive Boosting (AdaBoost)

The basic idea of the AdaBoost algorithm is to combine a sequence of weak learners through a weighted majority vote (or sum) to make classifications. It repeatedly updates the data by taking the previous weak learners’ mistakes into account. The basic steps of this algorithm can be explained as follows [30].

Given a classification training dataset $D=\left\{\left(x_{1},y_{1}\right),\left(x_{2},y_{2}\right),\cdots ,\left(x_{N},y_{N}\right)\right\}$, a strong classifier C(x) is generated by the following steps: 

Initialization of the weight value distribution of the training data,

(4) $$W_{1}=\left(w_{11},\cdots ,w_{1i},\cdots ,w_{1N},\right),\;w_{1i}=\frac{1}{N},i=1,2,\cdots ,N,\;m=1,2,\cdots ,M\;(m\;\mathrm{is}\;\mathrm{the}\;\mathrm{times}\;\mathrm{of}\;\;\mathrm{iteration})$$

Using the training dataset has the weight distribution $W_{m}$ to learn, and the basic classification $C_{m}\left(x\right)$ according to the Gini indexes of different influencing factors k is calculated. 

The classification error rate of $C_{m}\left(x\right)$ is calculated as follows

(5) $$e_{m}=P\left(C_{m}\left(x\right)\neq y_{i}\right)=\sum_{i=1}^{N}w_{\mathrm{mi}}I\left(C_{m}\left(x\right)\right)\neq y_{i}$$

The “amount of say”, $a_{m}$ of $C_{m}\left(x\right)$ is calculated according to its classification error $e_{m}$

(6) $$a_{m}=\frac{1}{2}\log\frac{1-e_{m}}{e_{m}}$$

The weight distribution based on the calculated “amount of say”, $a_{m}$, is updated

(7) $$W_{m}=\left(w_{m+1,1},\cdots ,w_{m+1,i},\cdots ,w_{m+1,N}\right)$$

(8) $$w_{m+1,i}=\frac{w_{\mathrm{mi}}}{Z_{m}}\exp\left(-a_{m}y_{i}C_{m}\left(x_{i}\right)\right)$$

where $Z_{m}$ is the normalization factor, which could make the sum of $W_{m}$ equal to 1.

The weighted sum of all the classifiers is calculated

(9) $$f\left(x\right)=\sum_{m=1}^{M}a_{m}C_{m}\left(x\right)$$

The final strong classifier can be expressed as

(10) $$C\left(x\right)=\mathrm{signf}\left(x\right)=\mathrm{sign}\left(\sum_{m=1}^{M}a_{m}C_{m}\left(x\right)\right)$$

### 3.4. Gradient Boosting Decision Tree (GBDT)

GBDT is a generalization of boosting to arbitrary differentiable loss functions. The motivation is to combine several weak models to produce a powerful ensemble [31]. Assume that F(x) is an approximation function of the dependent variable y based on a set of independent variables x. F(x) can be expressed as $F\left(x\right)=\sum_{m=1}^{M}\gamma_{m}h_{m}(x)$, where $h_{m}\left(x\right)$ are the basis functions, which are usually called weak learners in the context of boosting. The loss function can be defined as, $L\left(y,F\left(x\right)\right)=\log\left(1+e^{-\mathrm{yF}\left(x\right)}\right)$.

Similar to other boosting algorithms, GBDT builds the additive model in a greedy fashion:

(11) $$F_{m}\left(x\right)=F_{m-1}\left(x\right)+\gamma_{m}h_{m}\left(x\right)$$

where the newly added tree $h_{m}$ tries to minimize the loss L, given the previous ensemble $F_{m-1}\left(x\right)$:

(12) $$h_{m}=\underset{h}{\arg\;\min}\sum_{i=1}^{n}L\left(y_{i},F_{m-1}\left(x_{i}\right)+h\left(x_{i}\right)\right)$$

The initial model F_0_ is problem-specific; for the least-squares regression, one usually chooses the mean of the target values.

Gradient boosting attempts to solve this minimization problem numerically via steepest descent:

(13) $$F_{m}\left(x\right)=F_{m-1}\left(x\right)-\gamma_{m}\sum_{i=1}^{n}\nabla_{F}L\left(y_{i},F_{m-1}\left(x_{i}\right)\right)$$

where the step length $\gamma_{m}$ is chosen using the line search:

(14) $$\gamma_{m}=\underset{\gamma }{\arg\;\min}\sum_{i=1}^{n}L\left(y_{i},F_{m-1}\left(x_{i}\right)-\gamma \frac{\partial L\left(y_{i},F_{m-1}\left(x_{i}\right)\right)}{\partial F_{m-1}\left(x_{i}\right)}\right)$$

### 3.5. Mean Decrease Impurity (MDI) 

In ML-based classification tree methods, the impurity of the classification results from a tree node can be measured by the Gini index. Like entropy, the basic idea is to gauge the disorder of a grouping by the target variable. The Gini index for a node t in a single tree can be calculated using the following equation:

(15) $$G\left(t\right)=1-\sum_{i=1}^{m}p^{2}(i|t)$$

where G(t) denotes the Gini index for the node t; i represents the number of classes; $p^{2}(i|t)$ represents the estimated class probabilities.

The Gini index can be used to evaluate the importance of the independent variables. Generally, the impurity (measured by Gini index) decreases from each independent variable in a randomized tree, and the decrease in impurity can be averaged across trees to determine the importance of the variables [32]. This is known as the mean decrease impurity (MDI). The more an independent variable decreases the impurity, the more important the variable is. In this study, MDI was used to rank the importance of risk factors that contribute to the severity of large truck crashes. 

## 4. Result Analysis

### 4.1. Identified AK Crash Contributing Factors

As mentioned before, this study was to compare the performance of ML-based classification tree models with the regression model in terms of the models’ capabilities in identifying the crash severity contributing factors and analyzing their impacts. For this purpose, a mixed logit model was first developed and served as the benchmarking model. The results of the developed mixed logit model are presented in Table 3. It can be seen that eighteen independent variables have significant impacts (*p*-value < 0.005) on the dependent variable (y = 1 if it is an AK level crash; y = 0 otherwise). Among them, the coefficients of three independent variables are random across the observations because they yield statistically significant standard deviations (see the values in the parentheses). The positive (negative) coefficients mean that when the value of the independent variable increases, the probability that a large truck crash is an AK level crash will increase (decrease). The impacts of these eighteen independent variables are discussed in detail in the “Impacts of the identified contributing factors” section later on. Note that the lower the *p*-value of the estimated coefficient, the higher the significance level of this variable. Thus, the importance of the factors can be ranked according to their *p*-values.

Meanwhile, the ML classification tree models, i.e., RF, AdaBoost, and GBDT, were also developed to identify the crash risk factors that will significantly affect the severity of large truck crashes based on the estimated Gini index. The ML classification tree models were implemented in Python using the scikit-learn (0.19.1) package. For the development of the classification tree models, the number of trees for each type of model needs to be determined based on the model performance. In this study, a prediction accuracy measure, namely the receiver operating characteristic (ROC) curve, was used for evaluating the model performance. A ROC curve is a graphical plot for demonstrating the discriminant ability of a binary classifier system as its threshold is varied. The results showed that 200, 400, 200 trees are the optimal number of trees to achieve minimum misclassification for the RF, AdaBoost, and GBDT models, respectively. Based on the developed models, the importance of different independent variables can be assessed according to their MDI measures.

**Figure 1:**
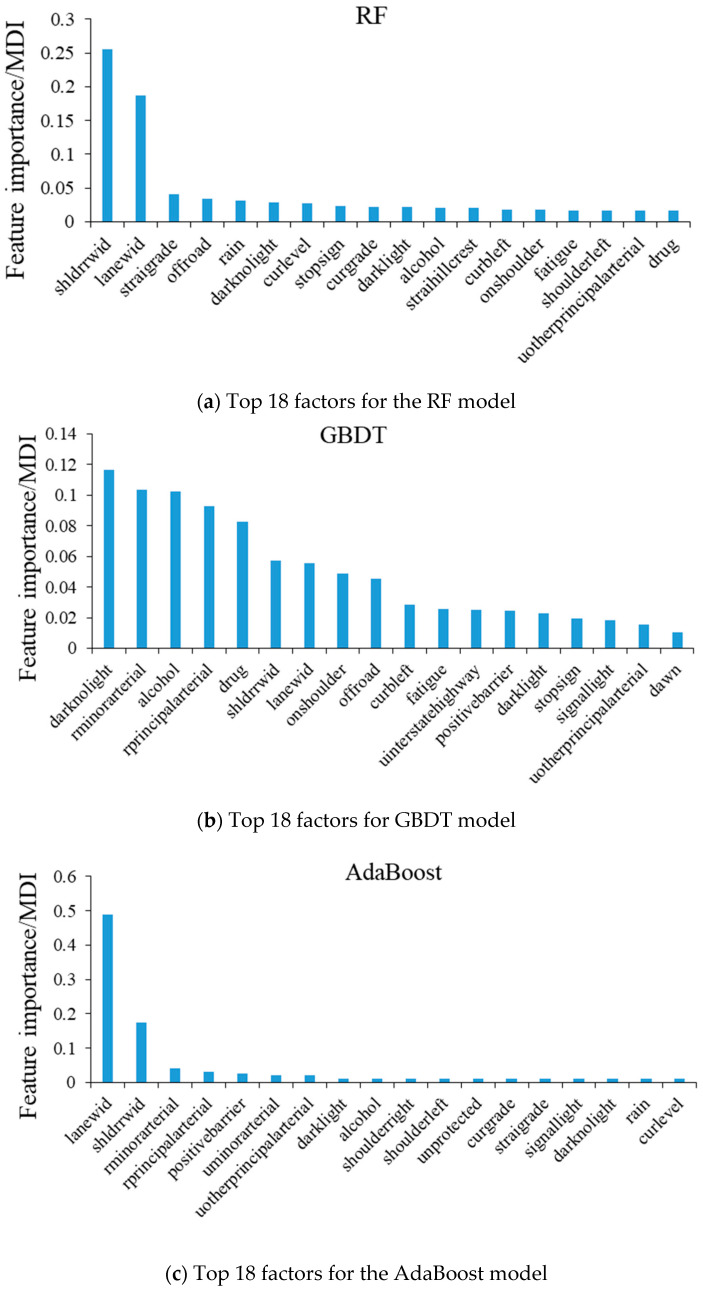
Top 18 factors identified by the three machine learning (ML) models. RF, random forest; GBDT, gradient boost decision tree; AdaBoost, adaptive boosting.

Since the mixed logit modeling results (see Table 3) indicate that there are 18 significant crash risk factors, for comparison purposes, only the top 18 most important risk factors were selected for each ML model, as presented in Figure 1.

**Figure 2:**
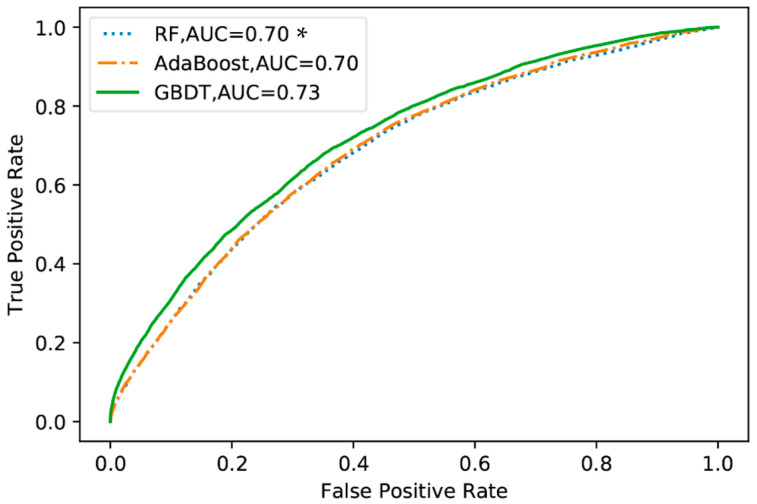
Receiver operating characteristic (ROC) curves of three ML models. * Note: AUC = area under the ROC curves.

To compare the results of ML models with the mixed model, Spearman’s rank correlation analysis was conducted between the importance ranks of the factors identified by the ML models and their importance ranks based on the *p*-values of their coefficients in the mixed logit model. The following are the results:

The correlation analysis results in Table 4 implies that, compared with the RF and AdaBoost models, the results of the GBDT model are more correlated with the results of the mixed logit model. Therefore, if we choose an ML method for preselecting the independent variables for the mixed logit model, the GBDT model should be chosen because it can better identify important variables for the mixed logit model than the other two models. In addition, according to Figure 2, the area under the ROC curves (AUC) of the GBDT model is greater than those of RF and Adaboost, which indicates that the GBDT model has better prediction performance than the other two models. Thus, the GBDT model is selected for comparing with the mixed logit model for analyzing the impacts of the identified crash contributing factors. 

### 4.2. Analysis of the Impacts of the Identified Crash Contributing Factors 

For the mixed model, the impacts of factors were determined based on the estimated coefficients of the independent variables. The positive coefficient indicates that the independent variable has a positive effect on the dependent variable, and vice versa. For the GBDT model, partial dependence plots were used for analyzing the directions of impacts. 

### 4.3. Partial Dependence Plots of GBDT Model 

**Figure 3:**
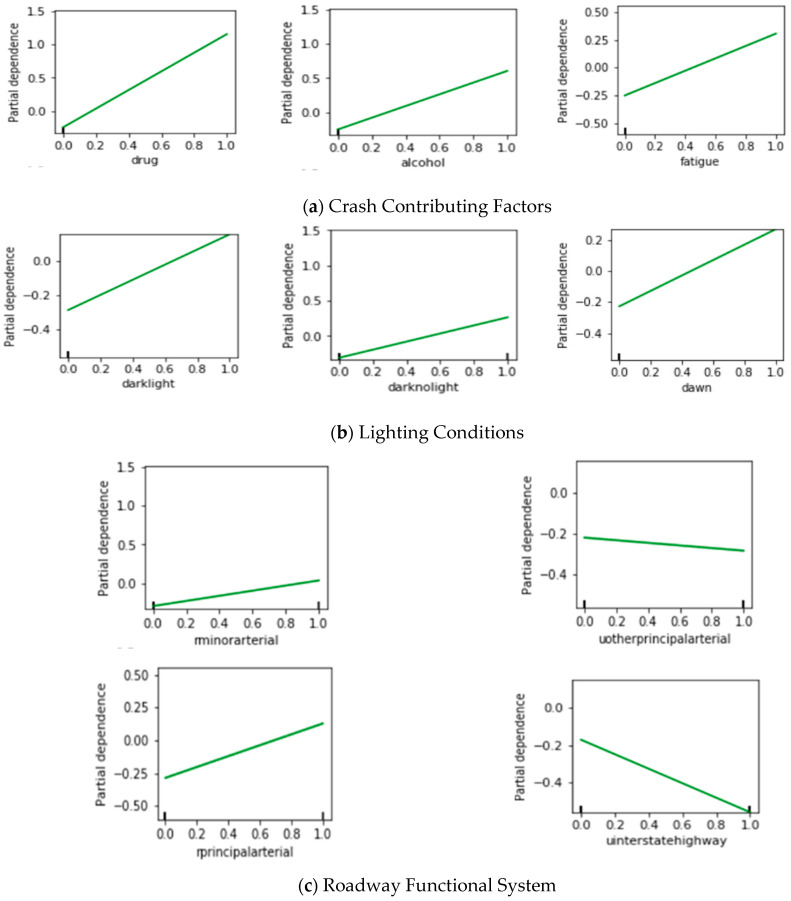

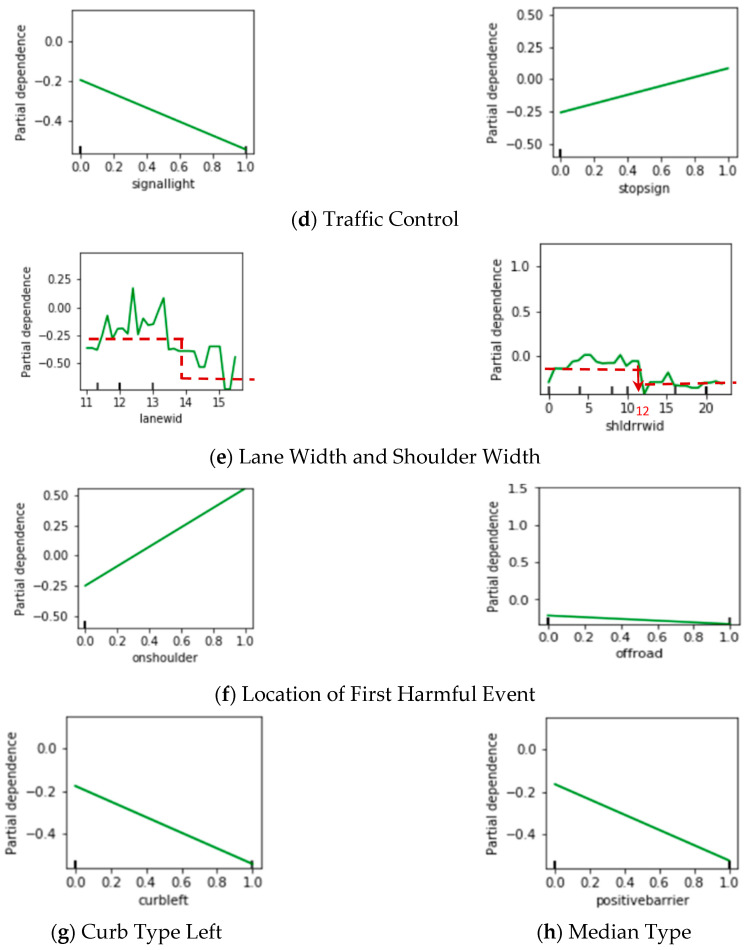
Partial dependence plots of different independent variables: (**a**)–(**h**) indicate different variable categories.

To examine complex risk patterns of large trucks, partial dependence plots were constructed. A partial dependence shows the dependence between the outcomes (crash severity levels) and a set of crash contributing factors; it indicates the marginal effect the factors have on the predicted outcomes [31]. The partial dependence plots of the top 18 factors are presented in Figure 3. 

From these partial dependence plots, the impacts of the dependent variable on the independent variable can be analyzed according to the direction of the plotted lines. More specifically, if the partial dependence increases with the increase in the value of an independent variable, this independent variable has a positive effect on the dependent variable, and vice versa. In addition, the slopes of the plotted lines indicate the magnitudes of the impacts. Comparing with the mixed logit model, the advantage of the GBDT model is that it can reveal nonlinear relationships between the numerical independent variables and the dependent variables. For example, as shown in Figure 3e, the impact of lane width (lanewid) does not decline in a straight line; instead, it roughly shows a piecewise decline with spikes at some points that may be due to some random factors. 

### 4.4. Comparison of the Mixed Logit Model and GBDT Model

To further compare the results of the mixed logit model and the GBDT model, the factors identified by these two models and their impacts on the dependent variable are summarized and listed together in Table 5.

Table 5 consists of three parts. The top part lists the nine factors that were identified by both the mixed logit model and the GBDT model. These factors belong to six categories, including “Crash contributing factors”, “Roadway functional system”, “Traffic control”, “Curb type left”, “Median type”, and “Location of first harmful event”. First, the signs of the coefficients of the mixed logit model are consistent with the directions of the partial dependence plots of the GBDT model shown in Figure 3. It means that the directions of the impacts for these factors are consistent according to the results of both models. In addition, both models identified “driver under the influence of a drug” as the important contributing factor to the AK level large truck crashes. 

In the mid part of Table 5, it lists the nine factors that were only identified by the mixed logit model but not the GBDT model. These factors belong to three categories, including “Weather characteristics”, “Road alignment”, and “Median type”. Note that, for the category “Weather characteristics”, the results of the mixed logit model showed that the weather conditions of “snowing”, “sleet”, “raining”, and “severe crosswinds” would all decrease the chance of a severe large truck crash, which is not reasonable and contradicts with the findings of some previous studies [9,33].

In the lower part of Table 5, it lists the nine factors that were only identified by the GBDT model but not the mixed logit model. These factors belong to five categories. More factors related to lighting conditions and crash contributing factors were identified. Overall, the influencing directions of these factors are all reasonable and explainable. It is worth mentioning that the GBDT model can identify the numerical factors like lane wide (lanwid) and shoulder wide (shldrrwid), while all the factors identified by the mixed model are category variables. It is because, for the numerical factors, they very likely to have nonlinear impacts on the dependent variable, and this type of impact can be hardly identified by the mixed logit regression model, which is built upon a linear link function between the x and y. 

Overall, the GBDT model identified more factors related to lighting conditions and crash contributing factors, while the mixed logit model identified more factors related to weather characteristics and roadway geometric characteristics. About half of the factors were identified by both models, and for these factors, the results of both models are consistent. In the following section, the impacts of the identified factors are discussed in detail. 

### 4.5. The Impacts of the Identified Contributing Factors

As shown in Table 5, there was a total of 27 factors identified either by the mixed logit model or by the GBDT model. In this section, the impacts of these 27 factors are discussed according to the modeling results by categories. First, the factors identified by the GBDT model were analyzed according to the order of the categories presented in Figure 3. After that, the categories that were only identified by the mixed logit model but not by the GBDT model were analyzed according to the order of the categories presented in Table 5.

(1) Crash Contributing Factors

Three crash contributing factors—“driver under the influence of a drug”, “driver under the influence of alcohol”, and “driver under the influence of fatigue”—were identified as the important factors by the GBDT model. Among them, the factors “driver under the influence of a drug” and “driver under the influence of fatigue” were also identified by the mixed logit model as the significant factors. Overall, these three factors all have positive impacts on the dependent variable, which means that these three factors all tend to result in severe large truck crashes. This is reasonable. Fatigue, alcohol, and drug can affect human brains and other parts of the nervous system and, thus, slow down a person’s activity or responsiveness. Therefore, truck drivers’ reactions and decision-making can be greatly affected if they are under the influence of these factors, leading to risky driving situations [34]. In terms of the magnitude of the impacts, according to the estimated variable coefficients of the mixed logit model, the coefficient for the “driver under the influence of a drug” is 2.0215, and the coefficient for the “driver under the influence of fatigue” is 0.7642. This indicates that the impacts of “driver under the influence of a drug” are greater than the impacts of “driver under the influence of fatigue”. In addition, according to the slopes of the partial dependence plots presented in Figure 3a, it can be seen that the “driver under the influence of a drug” has the biggest impacts, followed by “alcohol” and “fatigue”. Overall, the results of both models are consistent, while GBDT can identify more driving under influencing (DUI)-related factors.

(2) Lighting Conditions

Lighting conditions factors were only identified by the GBDT model but not the mixed logit model. They are “incident occurred when dark not lighted”, “incident occurred when dark but lighted”, and “incident occurred when dawn”. These three factors all have positive impacts on the dependent variable, which means that they will increase the chance of an AK level large truck crashes. In general, crashes that occur at dawn or night (with light or without light) tend to be more severe than crashes that occur during the day. It is because that reduced visibility at night has an adverse effect on truck drivers’ reaction time.

(3) Roadway Functional System

Four roadway functional system factors—“urban interstate highway”, “urban other principal arterial”, “rural minor arterial”, and “rural principal arterial”—were identified as the important factors by the GBDT model. Among them, the factors “urban interstate highway” and “urban other principal arterial” were also identified by the mixed logit model as significant factors. The results of both models are consistent, and all indicate that “urban interstate highway” and “urban other principal arterial” have negative impacts on the dependent variable, which means that there is less chance of an AK level large truck crashes on these two types of roadway. It is reasonable because urban roadways usually have better traffic and access control and are, therefore, generally safer than rural roads. In addition, according to the slopes of the partial dependence plots presented in Figure 3c, large truck crashes on a rural principal arterial are most likely to be severe crashes, followed by those on rural minor arterials. These results are also reasonable because truck drivers usually drive at a higher speed on rural roadways, especially on rural principal arterials, which increases the risk of severe crashes.

(4) Traffic Control

Two traffic control factors—“stop sign” and “signal light”—were identified as the important factors by the GBDT model and were also identified by the mixed logit model as significant factors. The results of both models are consistent, and all indicate that “stop sign” has positive impacts on the dependent variable, while “signal light” has negative impacts on the dependent variable. It is reasonable because stop signs usually are installed at rural intersections with high-speed limits, where the risk of severe truck crashes is high, and traffic signals can prevent the severe truck crashes by clearly assigning the right-of-way to the vehicles from different directions to keep traffic in order.

(5) Lane Width and Shoulder Width

The factors—“ lane width” and “right shoulder width”—were identified by the GBDT model but not by the mixed logit model. According to Figure 3e, both the “lane width” (lanewid) and “right shoulder width” (shldrrwid) plots show a piecewise decline. These results are reasonable. According to Zegeer et al. (1980), a wider lane width reduces the risk of the run-off-road and opposite-direction crashes, which are usually AK level crashes [35]. From the partial dependence plot of the “right shoulder width”, it can be seen that there is a sudden drop at the point of 12ft shoulder width. According to Federal Highway Administration (FHWA) safety acquirement [36], a paved shoulder width of 12 feet is required for the interstate highway with truck traffic exceeding 250 directional design hour volume (DDHV). Note that in roadway segments where the shoulder width is less than 12ft, a stalled truck cannot be safely refuged on the shoulder, which significantly increases the risk of severe truck crashes. In addition, wider shoulder width allows vehicles to pull off the road in emergencies and have clearance from traffic, which brings the drivers a high perception of safety [37].

(6) Types of Curbs

The existence of left curbs (curbleft) was identified in both models with consistent negative impacts. When a crash occurs, curbs can help prevent the large truck from running off the road. These preventive measures can lead to less severe crashes.

(7) Location of First Harmful Event

Two locations of first harmful event factors—“crash occurred off-road” and “crash occurred on the shoulder”—were identified as the important factors by the GBDT model, and “crash occurred off-road” was also identified by the mixed logit model as a significant factor. According to both models, the factor “crash occurred off-road” has negative impacts on the dependent variable. Since, in this category, we used “the large truck crashes that occurred on the road” as the baseline variable, it can be seen that large truck crashes that occurred off the road usually are less severe than those that occurred on the road. It is because off-road crashes usually are due to a truck hitting a fixed object (like a tree) rather than hitting another moving vehicle, which usually results in less injury and fatality. According to the GBDT model, “crash occurred on the shoulder” has positive impacts on the dependent variable, which means that a large truck crash occurred on the shoulder tend to be more severe than that occurred on the road. It is because this type of crash usually is the result of a large truck hitting a stalled vehicle on the shoulder or another moving vehicle hitting a stalled large truck on the shoulder, which often causes incapacitating injuries or fatalities.

(8) Median Type

“Median type is positive barrier” was identified by both the mixed logit model and the GBDT model as a negative influencing factor, which indicates that barriers installed on the median can help reduce the risk of severe large truck crashes. In addition, “median type is unprotected” and “median type is one-way pair” were only identified by the mixed logit model as a negative influencing factor. Note that the baseline variable in the “median type” category is “no median”. Thus, these results indicate that the roadways with unprotected median or one-way roadway have less risk for AK level large truck crashes than the roadway with no median. These results are also reasonable. It is because, on these two types of roadway, the traffic from two opposite directions are better separated than that on a roadway with no median. As a result, when a crash occurs, there is less chance for the large truck running into the opposite direction of the road and resulting in severe crashes. These results are also consistent with the findings in the literature [38].

(9) Weather Characteristics

Four weather characteristic factors—“snowing”, “sleet”, “severe crosswinds”, and “raining”—were only identified by the mixed logit model but not the GBDT model. These four weather characteristic factors all have negative impacts on the dependent variable. These results are not reasonable and contradict the findings in the literature. For example, Malin et al. (2019) found that accident risks increase under bad road weather conditions, especially for icy rain and slippery road conditions [33].

(10) Road Alignment

Three road alignment factors—“straight grade”, “curve grade”, and “curve level”—were only identified by the mixed logit model but not the GBDT model. Overall, these three factors all have positive impacts on the dependent variable. As compared to a straight level road segment, curved road, grade road, and curved with grade road are more likely to result in severe large truck crashes. This is because large truck drivers have limited sight distance when approaching the road segments with curves, slopes, or curved slopes. In addition, it is difficult for a large truck driver to apply brakes sufficiently on a curved grade road. 

## 5. Conclusions and Recommendations

In this study, three classification tree-based ML models, i.e., RF, AdaBoost, and GBDT models, and a mixed logit model were developed for analyzing the severity of the large truck crashes. We compared the performance of these models in terms of their capabilities in identifying the right risky factors and analyzing their impacts. The following are some key findings and some corresponding recommendations:

(1) The importance rank of the factors identified by the GBDT model is more correlated with the significant level of the factors identified by the mixed logit model than those of the RF and AdaBoost models. This result indicates that the GBDT model is a better choice for preselecting the variables for the mixed logit model.
(2) The partial dependence plots of the GBDT model can be used effectively in deriving the direction and magnitude of the impacts of the identified factors.
(3) For the factors that are identified by both GBDT and mixed logit models, the directions of the impacts of these factors are consistent according to the results of both models.
(4) For the factors that are only identified by the GBDT model, the directions and magnitudes of their impacts are all reasonable and explainable according to the partial dependence plots of the GBDT model.
(5) For the factors only identified by the mixed logit model, it is found that influencing directions of the weather characteristic factors are not reasonable.
(6) All the factors identified by the mixed model are categorical variables, while the GBDT model can also identify numerical factors like lane width (lanwid) and right shoulder width (shldrrwid), which have nonlinear impacts on the dependent variable.
(7) According to the partial dependence plots of GBDT, driving under the influence of drugs, alcohol, and fatigue are important contributing factors to the severity of large truck crashes. In addition, the existence of curbs, medians, and lanes and shoulders with sufficient width can effectively prevent severe large truck crashes.

The results of this study can help researchers in applying the ML-based methods for crash severity analysis in the future. It also can help traffic engineers better understand the contributing factors to the severity of large truck crashes and thereby choosing the most effective countermeasures for preventing severe large truck crashes. For example, fatigue, alcohol, and drug are identified as top contributing factors. For preventing driving under the influence of drugs, alcohol, and fatigue, in-vehicle driver drowsiness detection, alcohol detection, and drug detection systems could be implemented. Improving law enforcement and disseminating knowledge of traffic regulations to road users will also help. In addition, adequate lighting should be installed in the road segments with a high percentage of trucks to improve the roadway visibility at night. Furthermore, roads can be better designed by installing proper curbs, medians, and shoulders and lanes with sufficient widths to ensure the safe moving of large trucks. 

In this study, due to the data quality limitations, some potential contributing factors, such as the number of lanes and speed limit at the crash sites, have not been included in the developed models. In the future, more data need to be collected to include these variables to refine the modeling results. Besides, as shown in Table 2, the collected traffic crash data is very unbalanced as some of the categories only have very few cases. To address the imbalanced data problem, some resampling techniques will be employed to balance the data before model development in future studies. Furthermore, this research only considered four modeling methods. In the future, more methods should be considered to obtain a more comprehensive understanding of different models’ capabilities in crash severity analysis. 

## Figures and Tables

**Table 1:** Variables and descriptions. (* Baseline variable for each category).

**Traffic Control**		**Lighting Conditions**	
none *	1 if no traffic control, 0 otherwise (baseline)	daylight *	1 if incident occurred when daylight, 0 otherwise (baseline)
stopsign	1 if traffic control is stop sign, 0 otherwise	darknolight	1 if incident occurred when dark not lighted, 0 otherwise
signallight	1 if traffic control is signal light, 0 otherwise	dawn	1 if incident occurred when dawn, 0 otherwise
yieldsign	1 if traffic control is yield sign, 0 otherwise	darklight	1 if incident occurred when dark but lighted, 0 otherwise
flashinglight	1 if traffic control is flashing light, 0 otherwise	dusk	1 if incident occurred when dusk, 0 otherwise
**Roadway Functional System**		**Road Alignment**	
rintersatehighway *	1 if rural interstate highway, 0 otherwise (baseline)	strailevel *	1 if road alignment is straight level, 0 otherwise (baseline)
uinterstatehighway	1 if urban interstate highway, 0 otherwise	straigrade	1 if road alignment is straight grade, 0 otherwise
rprincipalarterial	1 if rural principal arterial, 0 otherwise	straihillcrest	1 if road alignment is straight hillcrest, 0 otherwise
uotherprincipalarterial	1 if urban other principal arterial, 0 otherwise	curlevel	1 if road alignment is curve level, 0 otherwise
uminorarterial	1 if urban minor arterial, 0 otherwise	curgrade	1 if road alignment is curve grade, 0 otherwise
rminorarterial	1 if rural minor arterial, 0 otherwise	curhillcrest	1 if road alignment is curve hillcrest, 0 otherwise
**Median Type**		**Location of First Harmful Event**	
mediannone *	1 if no median, 0 otherwise (baseline)	onroad *	1 if crash occurred on road, 0 otherwise (baseline)
unprotected	1 if median type is unprotected, 0 otherwise	onshoulder	1 if crash occurred on shoulder, 0 otherwise
positivebarrier	1 if median type is positive barrier, 0 otherwise	onmedian	1 if crash occurred on median, 0 otherwise
onewaypair	1 if median type is one-way pair, 0 otherwise	offroad	1 if crash occurred off-road, 0 otherwise
**Shoulder Type Left**		**Shoulder Type Right**	
shoulderlnone *	1 if no left shoulder, 0 otherwise (baseline)	shoulderrnone *	1 if no right shoulder, 0 otherwise (baseline)
shoulderleft	1 if left shoulder exists, 0 otherwise	shoulderright	1 if right shoulder exists, 0 otherwise
**Curb Type Left**		**Curb Type Right**	
curblnone *	1 if no left curb, 0 otherwise (baseline)	curbrnone *	1 if no right curb, 0 otherwise (baseline)
curbleft	1 if left curb exists, 0 otherwise	curbright	1 if right curb exists, 0 otherwise
**Weather Characteristics**		**Crash Contributing Factors**	
clear *	1 if clear, 0 otherwise (baseline)	fatigue	1 if driver under the influence of fatigue, 0 otherwise
rain	1 if raining, 0 otherwise	drug	1 if driver under the influence of a drug, 0 otherwise
snow	1 if snowing, 0 otherwise	alcohol	1 if driver under the influence of alcohol, 0 otherwise
blowing	1 if blowing sand, 0 otherwise		
fog sleet severcrosswinds	1 if fog, 0 otherwise 1 if sleet, 0 otherwise 1 if severe crosswinds, 0 otherwise	**Lane Width and Shoulder Width**	
		lanewid shldrrwid	The width of travel lanes in feet The width of the right shoulder in feet

**Table 2:** Distribution of the variables.

Variable	Crash Injury Severity		Total	Percent	Variable	Crash Injury Severity		Total	Percent
	Non-AK Crash	AK Crash				Non-AK Crash	AK Crash		
**Traffic Control**					**Light Characteristics**				
none	9275	395	9670	11.35%	daylight	59,034	3324	62,358	73.20%
stopsign	4392	452	4844	5.69%	darknolight	11,129	1383	12,512	14.69%
signallight	10,952	373	11,325	13.29%	dawn	1085	124	1209	1.42%
yieldsign	2016	123	2139	2.51%	darklight	7455	542	7997	9.39%
flashinglight	509	51	560	0.66%	dusk	708	42	750	0.88%
**Location of First Harmful Event**					**Median Type**				
onroad	65,535	4344	69,879	82.03%	mediannone	29,965	2816	32,781	38.48%
onshoulder	1015	169	1184	1.39%	unprotected	21,286	1482	22,768	26.73%
onmedian	2818	180	2998	3.52%	positivebarrier	24,565	1014	25,579	30.03%
offroad	10,345	747	11,092	13.02%	onewaypair	483	11	494	0.58%
**Roadway Functional System**					**Weather Characteristics**				
uinterstatehighway	21,666	851	22,517	26.43%	clear	69,592	4853	74,445	87.39%
rprincipalarterial	9642	1159	10,801	12.68%	rain	7470	410	7880	9.25%
uotherprincipalarterial	23,084	1050	24,134	28.33%	snow	752	19	771	0.91%
uminorarterial	4615	296	4911	5.77%	blowing	124	16	140	0.16%
rminorarterial	12,058	1453	13,511	15.86%	fog	738	97	835	0.98%
rintersatehighway	8679	631	9310	10.93%	sleet	675	25	700	0.82%
**Road Alignment**					severcrosswinds	368	18	386	0.45%
strailevel	58,853	3742	62,595	73.48%	**Crash Contributing Factors**				
straigrade	9424	715	10,139	11.90%	fatigue	1618	240	1858	2.18%
straihillcrest	2669	217	2886	3.39%	drug	206	99	305	0.36%
curlevel	4757	428	5185	6.09%	alcohol	862	241	1103	1.29%
curgrade	3191	291	3482	4.09%					
curhillcrest	615	37	652	0.77%					
**Shoulder Type Left**					**Curb Type Left**				
shoulderlnone	12,463	559	13,022	15.29%	curblnone	66,710	4971	71,681	84.15%
shoulderleft	63,903	4543	68,446	80.35%	curbleft	9307	339	9646	11.32%
**Shoulder Type Right**					**Curb Type Right**				
shoulderrnone	9576	433	10,009	11.75%	curbrnone	66,427	4960	71,387	83.80%
shoulderright	66,732	4660	71,392	83.81%	curbright	8188	300	8488	9.96%

**Table 3:** Results of the mixed logit model.

Variable		Coeff. *	Std. Error	t	*p*-Value **
**traffic control**	signallight	−0.5801	0.0644	−9.01	<0.0001
	stopsign	0.2037	0.0587	3.47	0.0005
location	Offroad *^a^	−2.5980 (2.9134)	0.5779	−4.50	<0.0001(<0.0001)
weather	Rain *^a^	−1.2411 (1.7131)	0.3592	−3.45	0.0006(<0.0001)
	sleet	−0.8620	0.2479	−3.48	0.0005
	snow	−1.4201	0.2880	−4.93	<0.0001
	severcrosswinds	−0.7184	0.3243	−2.22	0.0267
road alignment	straigrade	0.1569	0.0488	3.21	0.0013
	curlevel	0.2380	0.0669	3.56	0.0004
	curgrade	0.3068	0.0800	3.83	0.0001
functional system	uinterstatehighway	−0.7046	0.0618	−11.40	<0.0001
	uotherprincipalarterial	−0.5464	0.0480	−11.39	<0.0001
curb left	curbleft	−0.6472	0.0581	−11.13	<0.0001
median	positivebarrier	−0.5434	0.0548	−9.91	<0.0001
	Unprotected *^a^	−1.8760 (−2.2862)	0.3472	−5.40	<0.0001(<0.0001)
	onewaypair	−0.6444	0.3111	−2.07	0.0383
factors	drug	2.0215	0.1466	13.79	<0.0001
	fatigue	0.7642	0.0935	8.18	<0.0001
Intercept		−2.1065	0.0278	−75.85	<0.0001
Log likelihood	−19,352 (df = 22)				
Sample size	85,184				

* Parentheses indicate the standard deviations of the random coefficient estimates. ** Parentheses indicate the *p*-value of the standard deviations of the random coefficient estimates. *^a^ Denotes random parameters.

**Table 4:** Spearman’s rank correlation coefficients of factors’ importance rank.

	Importance Ranks Based on the MDI *^2^ Measures of the ML *^3^ Models		
Importance ranks based on the *p*-values of the mixed logit model	RF *^4^ model	GBDT *^5^ model	AdaBoost *^6^ model
	−0.059 (0.817 *^1^)	0.738 (0.001 *^1^)	0.152 (0.548 *^1^)

*^1^ The significance level of the estimated correlation coefficient, *^2^ MDI denotes Mean Decrease Impurity. *^3^ ML denotes Machine Learning. *^4^ RF denotes Random Forest. *^5^ GBDT denotes Gradient Boost Decision Tree. *^6^ AdaBoost denotes Adaptive Boosting.

**Table 5:** Comparison of the mixed logit model and the GBDT model.

Variable Category	Variables’ Descriptions	Mixed Logit			GBDT		
		Significant Factors (*p*-Value <5%)	Signs of β	Ranked by *p*-Value	Important Factors (Top 18)	Direction of Partial Dependence	Ranked by MDI
Crash contributing factors	**1 if driver under the influence of a drug, 0 otherwise**	**drug**	**+**	**1**	**drug**	+	**5**
	1 if driver under the influence of fatigue, 0 otherwise	fatigue	+	7	fatigue	+	11
Roadway functional system	1 if urban interstate highway, 0 otherwise	uinterstatehighway	**–**	2	uinterstatehighway	**–**	12
	1 if urban other principal arterial, 0 otherwise	uotherprincipalarterial	**–**	3	uotherprincipalarterial	**–**	17
Traffic control	1 if traffic control is stop sign, 0 otherwise	stopsign	+	14	stopsign	+	15
	1 if traffic control is signal light, 0 otherwise	signallight	**–**	6	signallight	**–**	16
Curb type left	1 if left curb exists, 0 otherwise	curbleft	**–**	4	curbleft	**–**	10
Median type	1 if median type is positive barrier, 0 otherwise	positivebarrier	**–**	5	positivebarrier	**–**	13
Location of first harmful event	1 if crash occurred off-road, 0 otherwise	offroad	**–**	10	offroad	**–**	9
Weather characteristics	**1 if snowing, 0 otherwise**	**snow**	**–**	**9**			
	**1 if sleet, 0 otherwise**	**sleet**	**–**	**13**			
	**1 if raining, 0 otherwise**	**rain**	**–**	**15**			
	**1 if severe crosswinds, 0 otherwise**	**severcrosswinds**	**–**	**17**			
Road alignment	1 if road alignment is straight grade, 0 otherwise	straigrade	**+**	16			
	1 if road alignment is curve level, 0 otherwise	curlevel	**+**	12			
	1 if road alignment is curve grade, 0 otherwise	curgrade	**+**	11			
Median type	**1 if median type is one-way pair, 0 otherwise**	**onewaypair**	**–**	**18**			
	1 if median type is unprotected, 0 otherwise	unprotected	**–**	8			
Lighting conditions	1 if incident occurred when dark not lighted, 0 otherwise				darknolight	**+**	1
	1 if incident occurred when dark but lighted, 0 otherwise				darklight	**+**	14
	1 if incident occurred when dawn, 0 otherwise				dawn	**+**	18
Crash contributing factors	1 if driver under the influence of alcohol, 0 otherwise				alcohol	**+**	3
**Lane width and shoulder width**	**The width of the right shoulder in feet**				**shldrrwid**	**–**	**6**
	**The width of travel lanes in feet**				**lanewid**	**–**	**7**
Location of first harmful event	1 if crash occurred on shoulder, 0 otherwise				onshoulder	**+**	8
Roadway functional system	1 if rural minor arterial, 0 otherwise				rminorarterial	**+**	2
	1 if rural principal arterial, 0 otherwise				rprincipalarterial	+	4
